# Neph1-Robo mechanical anchoring stabilizes neurite branch orientation independently of receptor-specific intracellular signaling

**DOI:** 10.21203/rs.3.rs-11121715/v1

**Published:** 2026-10-14

**Authors:** Yun-Hsien Lin, Chun-Hao Chen

## Abstract

Neurite morphogenesis is classically viewed as a signal-driven process in which extracellular cues activate intracellular pathways to direct growth. Here, we identify a distinct mechanism in which adhesion preserves neurite architecture independently of canonical receptor signaling. Using axonal branching in *Caenorhabditis elegans* PVP interneurons, we show that neuronal SYG-1/Neph1 and epithelial SAX-3/Robo establish a transcellular interface that stabilizes branch orientation and may help resist physiological tissue deformation. This stabilization requires SYG-1 membrane tethering and extracellular domains, but not its cytoplasmic signaling domain, and can be reconstituted with cytoplasmically truncated SYG-1 and SAX-3. The canonical Robo ligand SLT-1 is required for this anchoring mechanism and promotes SYG-1/SAX-3-dependent cell association in vitro. Moreover, an unrelated synthetic adhesion pair partially substitutes for the native receptors and restores branch orientation. These findings reveal a signaling-independent structural role for classical guidance receptors in stabilizing neurite architecture within a deforming tissue environment.

## Introduction

Precise neural connectivity depends on the accurate navigation and positioning of developing neurites. Growth cones integrate diverse extracellular cues, including secreted guidance molecules such as Slit and membrane-tethered cell adhesion molecules (CAMs), through receptors that typically engage intracellular signaling pathways to remodel the cytoskeleton and direct neurite growth [1, 2, 3, 4]. In parallel, mechanical forces contribute substantially to nervous system morphogenesis. Neurites respond to substrate stiffness, tension, and tissue deformation through mechanosensitive channels, adhesion complexes, and cytoskeleton coupled mechanotransduction pathways [5, 6, 7, 8, 9, 10], which in turn regulate growth cone migration, axon extension, and collateral branching [11, 12, 13, 14, 15, 16]. Adhesive interactions can also directly control neurite placement and stability. Differential SYG-1/SYG-2-mediated adhesion repositions developing neurites through a retrograde zippering mechanism between neurite shafts, demonstrating that adhesion itself can organize neurite placement during circuit assembly [16]. More recently, ligand interactions with the dendritic guidance receptor DMA-1 were shown to stabilize developing PVD dendrites by suppressing branch retraction and ectopic growth [17], illustrating that extracellular receptor-ligand interactions can separately regulate neurite growth and stabilization. However, these mechanisms operate primarily by controlling developmental neurite dynamics. Whether transcellular receptor complexes can instead serve as persistent structural anchors that preserve an established neurite position against subsequent tissue deformation, independently of receptor-specific intracellular signaling, remains largely unexplored.

A small number of developmental systems demonstrate that extracellular structures can function as physical tethers. In *C. elegans*, DEX-1 and DYF-7 anchor sensory dendrite endings during retrograde extension, while related extracellular matrix systems stabilize sensory structures in other organisms [18]. Zona pellucida domain extracellular matrices similarly contribute to tissue organization and mechanically couple specialized sensory structures [19], including vertebrate hair bundles [20], to surrounding extracellular matrices. These examples establish that extracellular scaffolds can stabilize neuronal structures mechanically. Whether conventional transmembrane adhesion or guidance receptors can similarly function as signaling-independent structural anchors, however, remains largely unknown.

SYG-1, the *C. elegans* ortholog of vertebrate Neph1, provides an intriguing candidate for such functional versatility. In its canonical role, SYG-1 forms a transcellular adhesion complex with SYG-2/Nephrin to specify synapse formation, while vertebrate Neph1 and nephrin form related adhesive complexes at the podocyte slit diaphragm [21, 22, 23, 24]. In these contexts, the cytoplasmic domain of SYG-1 contributes to intracellular signaling and cytoskeletal organization [21]. Similarly, SAX-3, the *C. elegans* ortholog of Robo receptors, is best known for mediating Slit-dependent axon guidance through conserved cytoplasmic motifs coupled to actin regulatory pathways [25, 26, 27]. Intriguingly, SYG-1 can also respond to a cleaved extracellular fragment of SAX-3 during glial migration. In that context, however, SYG-1 requires its cytoplasmic WIRS motif and downstream actin remodeling [28, 29]. Thus, the best characterized functions of both SYG-1 and SAX-3 ultimately engage intracellular signaling, leaving open whether their membrane-tethered extracellular architectures can themselves provide mechanical stabilization.

Here, using sexually dimorphic branching of the *C. elegans* PVP interneurons, we identify an unexpected structural function for SYG-1/Neph1 and SAX-3/Robo. We show that neuronal SYG-1 and epithelial SAX-3 establish an SLT-1-dependent transcellular interface that stabilizes PVP branch orientation against physiological tissue deformation. Remarkably, this function does not require the cytoplasmic signaling domains of either receptor and can be partially substituted using an unrelated synthetic adhesion pair. These findings reveal an unexpected structural role for classical guidance and adhesion receptors in preserving neurite architecture during tissue deformation.

### Results

## SYG-1 Is Required for Directional Stability of PVP Neurite Branches

PVP interneurons are bilaterally symmetric cholinergic neurons that extend longitudinal processes along the ventral nerve cord (Fig. 1a) [30]. In adult hermaphrodites, PVPs form stereotyped dorsal collateral branches near the vulval region, whereas they exhibit only sparse and irregular protrusions in males [31, 32]. Previous work demonstrated that this sexually dimorphic branching depends on insulin signaling and vulval epithelial cues and contributes to egg-laying behavior [32]. Given that neurite branching of nearby HSN neurons relies on neuron-epithelial adhesion [21], we initially hypothesized that PVP branch guidance might similarly involve the canonical SYG-1/Neph1 and SYG-2/Nephrin adhesion molecules.

Unexpectedly, loss of *syg-1* and *syg-2* caused only mild defects in PVP branch formation (Fig. 1b; Supplementary Fig. 1). Notably, *syg-1* mutants exhibited a highly penetrant and striking phenotype in which more than 90% of PVP branches projected in the opposite (ventral) direction (Fig. 1b, c). In contrast, branch projection was not defective in *syg-2* mutants (Fig. 1b, c). This phenotype reveals a previously unrecognized and SYG-2-independent role for SYG-1 in controlling PVP branch orientation.

### SYG-1 Contributes to Initial Directional Bias but Is Primarily Required to Maintain Branch Orientation

To determine whether branch mistargeting in *syg-1* mutants reflects defects in branch initiation or subsequent maintenance, we performed time-lapse imaging of PVP branch development beginning at the L4 stage, when PVP branches first emerge (Fig. 1d, e). While most PVP branches initially extended dorsally, around 20% of branches in the *syg-1* mutant projected ventrally from the outset, a much higher percentage than in wild-type animals (Fig. 1f). This result shows that SYG-1 contributes to directional bias during early branch targeting. Importantly, even branches that initially extended dorsally frequently reversed orientation and became ventrally displaced at later stages (Fig. 1e). Consistent with this observation, LifeAct-based time-lapse imaging revealed that actin enrichment at nascent branch sites was comparable between wild-type and *syg-1* mutants and was stably maintained at mature branch tips (Fig. 1d, e; Supplementary Fig. 2) [33]. These observations indicate that SYG-1 is dispensable for actin enrichment and branch formation, but contributes both to initial directional bias and, more prominently, to subsequent maintenance of branch orientation.

**Reproductive Tissue Deformation Promotes Branch Displacement in syg-1 Mutants**

The delayed reversal of initially correctly oriented branches suggested that mistargeting might result from passive displacement rather than active misguidance. Egg accumulation, ovulation, and particularly egg passage through the vulva impose recurrent mechanical perturbations on tissues surrounding the reproductive tract [34]. To reduce egg production and the associated reproductive tissue deformation, we treated animals with 5-fluorodeoxyuridine (FUdR) [35]. To distinguish the effects of reduced reproductive activity from nonspecific effects of FUdR treatment, we classified FUdR-treated *syg-1* mutants according to whether eggs were present at the time of scoring. Animals lacking eggs showed a striking reduction in reversed PVP branching, from approximately 90% to ~ 50%, whereas FUdR-treated animals that retained eggs exhibited a reversal frequency comparable to untreated *syg-1* mutants (Fig. 1g, h). Thus, suppression of branch displacement correlated with the absence of eggs rather than FUdR exposure itself. This supports the idea that reproductive activity promotes branch displacement, consistent with mechanical deformation contributing to branch reversal. Together with the delayed reversal of initially correctly oriented branches, these results support a model in which reproductive tissue deformation contributes to branch displacement when SYG-1-mediated stabilization is lost.

### SYG-1 Functions Cell-Autonomously and Requires Only Its Membrane-Tethered Ectodomain

To localize SYG-1 functional sites, we performed tissue-specific rescue experiments. Pan-neuronal expression of SYG-1 fully restored correct branch orientation in *syg-1* mutants, whereas expression in vulval epithelial cells did not (Fig. 2a-c). Expression restricted specifically to PVP neurons was sufficient for rescue, while expression in HSN neurons was ineffective, demonstrating that SYG-1 acts cell-autonomously within PVP neurons (Fig. 2c).

To define the molecular requirements for this function, we performed domain-specific rescue experiments using truncated SYG-1 variants. SYG-1 comprises five extracellular immunoglobulin (Ig) domains and a cytoplasmic tail containing a WIRS motif that recruits actin regulators (Fig. 2a) [36]. Deletion of all extracellular immunoglobulin (Ig) domains, or removal of Ig1-2 alone, abolished rescue activity, whereas deletion of Ig4-5 had no effect (Fig. 2d, e). Strikingly, removal of the entire cytoplasmic tail, including the WIRS motif required for SYG-1 signaling in other contexts, did not impair rescue (Fig. 2f, g). In contrast, deletion of the transmembrane domain eliminated SYG-1 function (Fig. 2f, g). These results demonstrate that SYG-1 requires membrane tethering and its extracellular region to stabilize PVP branch orientation, whereas its receptor-specific cytoplasmic signaling domain is dispensable.

### SAX-3/ROBO Acts from the Vulval Epithelium in the Same Genetic Pathway as SYG-1

Because vulval epithelial cues are necessary to promote PVP branching, we next sought to identify vulval-expressed partners that cooperate with neuronal SYG-1. Screening candidate genes derived from the vulval transcriptome revealed that loss of *sax-3*, which encodes the homolog of Robo in *C. elegans*, substantially increased the frequency of reversed PVP branches (Fig. 3a-c) [37]. The *syg-1 sax-3* double mutant showed no enhancement of the phenotype, consistent with these two genes acting in the same genetic pathway (Fig. 3d). Expression of SAX-3 under its endogenous promoter fully rescued the branching defect in *sax-3* mutants (Fig. 3e). Because SAX-3 is expressed in the nervous system and vulva cells, we utilized tissue-specific promoters to drive SAX-3 expression in PVP and vulva cells, respectively, to investigate their functional localization (Fig. 3e). Tissue-specific rescue experiments further revealed that SAX-3 expression in vulval epithelial cells, but not in PVP neurons, restored correct branch orientation (Fig. 3e). These findings indicate that SAX-3 acts in vulval epithelial cells to stabilize PVP branch orientation (Fig. 3f).

### SLT-1 promotes SYG-1/SAX-3-dependent transcellular adhesion

Our results showed that SYG-1 and SAX-3 acted in opposing cells, raising the possibility that these receptors form a complex via *trans*-interactions. Previous work showed that secreted SAX-3 can act as a ligand for SYG-1 in other developmental contexts, independently of *slt-1*, a canonical Robo ligand [28]. However, expression of secreted SAX-3 from CAN neurons failed to rescue the branching defect in *sax-3* mutants (Supplementary Fig. 3), implying that membrane-tethered SAX-3 is required. While our initial characterization excluded a role for *syg-2* in branch orientation, whether *slt-1* participates in this interaction remained unclear. Notably, loss of *slt-1*, encoding the canonical SAX-3 ligand, phenocopied the *sax-3* mutants. The *slt-1* phenotype was rescued by endogenous SLT-1 expression (Fig. 4a, b). Furthermore, the *slt-1 syg-1* double mutant showed no enhancement of reversed PVP branching, placing SLT-1 in the same genetic pathway (Fig. 4c).

In *Drosophila*, proteolytic cleavage of Slit has been shown to enhance the assembly of Robo1 with the adhesion molecule Dscam1, thereby promoting axonal growth [38]. Drawing on this paradigm, we hypothesized that SYG-1 and SAX-3 form an adhesive complex in an SLT-1-dependent manner. This model yields two testable predictions: (i) *slt-1* should be expressed in proximity to the vulval region, and (ii) SLT-1 should promote SYG-1/SAX-3-dependent cell association. To test the first prediction, we examined *slt-1* expression using a transcriptional reporter driven by a 4.5 kb upstream promoter fragment. We found that *slt-1* was expressed in primary vulval cells at the L4 stage, coinciding temporally and spatially with PVP branch formation and targeting (Fig. 4a). To test the second prediction, SYG-1ΔC- and SAX-3ΔC-expressing S2 cell populations were generated separately and subsequently mixed to assess cell-cell association (Fig. 4d). Untransfected S2 cells showed little spontaneous aggregation (Fig. 4e, f). Similarly, mixing of SYG-1ΔC and SAX-3ΔC had a comparable effect to mock treatment in cell aggregation. Strikingly, aggregation was markedly enhanced when the cells were incubated with conditioned medium from SLT-1-expressing cells (Fig. 4e, f). These results indicate that extracellular SLT-1 promotes the association between SYG-1 and SAX-3, supporting a model in which SLT-1 promotes formation or stabilization of the SYG-1/SAX-3 adhesive interface.

## Extracellular Adhesion Is Sufficient to Partially Restore PVP Branch Orientation

How do SYG-1 and SAX-3 regulate PVP branch targeting in the absence of cytosolic signaling domains? We considered two alternative models. In the first model, SYG-1 and SAX-3 form a transcellular adhesive complex whose physical linkage is sufficient to stabilize branch orientation (Fig. 5a). In the second model, SYG-1 acts as a membrane organizer that patterns the localization or activity of other surface receptors in PVP neurons to guide branch targeting indirectly (Fig. 5a).

To distinguish between these possibilities, we performed tissue-swapping experiments in *syg-1* mutants by expressing SYG-1 in vulval epithelial cells and SAX-3 in PVP neurons. To eliminate potential contributions from intracellular signaling, we additionally truncated the cytosolic domains of both proteins. This reciprocal expression paradigm partially restored branch orientation (Fig. 5b). Interestingly, this rescue was strictly dependent on SLT-1, suggesting that the SYG-1-SAX-3 interaction requires SLT-1 (Fig. 5c). Together, these results support a model in which the extracellular SYG-1/SAX-3 interface provides structural anchoring rather than relying primarily on receptor-specific downstream signaling, with formation or stabilization of this interface remaining SLT-1 dependent.

To directly test whether receptor-specific signaling is unnecessary and an unrelated extracellular adhesive interaction is sufficient to provide substantial rescue, we engineered a synthetic adhesion system in which transmembrane-tethered GFP nanobody (LaG16) was expressed in PVP neurons and a membrane-tethered GFP was expressed in vulval cells [39, 40]. This artificial adhesion interaction significantly rescued branch misguidance in *syg-1* mutants (Fig. 5d, e). These results suggest that an adhesive linkage across the neuron-epithelium interface can partially substitute for the native SYG-1/SAX-3 interaction, supporting the idea that physical adhesion, rather than receptor identity or canonical intracellular signaling, is sufficient to stabilize branch orientation.

### Correct PVP branch orientation is associated with efficient egg laying

What is the physiological significance of precise PVP branch targeting? Previous studies have shown that PVP branch formation modulates egg-laying behavior via serotonergic signaling [32]. We therefore asked whether mistargeting of PVP branches leads to functional defects in egg laying.

To address this, we analyzed *syg-1* mutants carrying a synthetic nanobody-GFP adhesive pair, which restores ~ 50% of correct PVP branch targeting. We quantified their egg-laying rates in food-free liquid medium supplemented with or without serotonin [41]. Animals were subsequently stratified based on their terminal PVP branch configuration (Fig. 6a). This setup thereby bypassed the major egg-laying promoting neurons, which exhibit axonal branching defects in *syg-1* mutants [21, 42]. As expected, in the absence of serotonin, egg laying was uniformly reduced in liquid conditions regardless of branch configuration. Notably, *syg-1* mutants in which PVP targeting was partially rescued by synthetic adhesion exhibited significantly higher egg-laying rates compared to animals with reversed branches (Fig. 6b). These results establish an association between correct PVP branch positioning and more efficient serotonin-evoked egg laying.

## Discussion

Neurite morphogenesis is classically understood as a signal-driven process in which extracellular cues are interpreted through intracellular signaling pathways that remodel the cytoskeleton and direct neurite growth. Our findings show that PVP branch position is actively preserved after outgrowth by an adhesive interface formed across the neuron-vulval epithelium boundary. Neuronal SYG-1/Neph1 and epithelial SAX-3/Robo cooperate with SLT-1 to maintain branch orientation as surrounding reproductive tissues deform. Remarkably, this stabilizing function can operate without the receptor-specific cytoplasmic domains and can be partially reproduced with an unrelated synthetic adhesion system. Thus, a minimal extracellular adhesion module can provide substantial structural stabilization of neurite architecture against tissue deformation.

Our results also support a model that SYG-1 and SAX-3 form a transcellular adhesive interface that stabilizes PVP neurite orientation across the tissue boundary. This organization parallels the well-characterized HSN synaptogenesis system, in which neuronal SYG-1 interacts with SYG-2/Nephrin on vulval epithelial guidepost cells to specify synapse position [21]. However, the functional outputs of extracellular adhesion can be strikingly different. Differential SYG-1/SYG-2 adhesion can actively reposition developing neurites through a retrograde zippering mechanism, whereas recent work on PVD dendrites showed that extracellular ligand engagement of DMA-1 stabilizes developing branches by limiting retraction and inappropriate growth [17]. In PVP neurons, the SYG-1/SAX-3 interface performs a further distinct function. Rather than primarily controlling branch growth or retraction, it preserves an already established branch position against subsequent deformation of surrounding reproductive tissues. Thus, extracellular adhesion can contribute to neural morphogenesis at multiple levels, including active neurite repositioning, stabilization of dynamic branches, and mechanical preservation of established architecture.

Interestingly, a potentially analogous structural role of Neph1 and Robo interactions may occur in the vertebrate kidney. Neph1 and nephrin are core components of the podocyte slit diaphragm, and Robo2 has been reported to associate with this protein network [43, 44]. Because the slit diaphragm is continuously exposed to mechanical stress generated by glomerular filtration, these interactions raise the possibility that Neph- and Robo-family proteins contribute not only to signaling but also to the mechanical stability of specialized cell-cell interfaces. Although whether an analogous structural mechanism operates in vertebrate tissues remains unknown, our findings suggest that the extracellular architecture of classical guidance and adhesion receptors may, in some contexts, provide structural resilience under mechanical load.

Our findings also clarify a conceptual distinction between mechanical anchoring and classical mechanotransduction. In canonical mechanotransduction, mechanical forces are converted into intracellular biochemical signals through mechanosensitive ion channels or cytoskeleton-coupled adhesion receptors, ultimately altering gene expression or cytoskeletal dynamics [7, 8, 9]. By contrast, in the SYG-1/SAX-3 system, the adhesive linkage itself appears to be the relevant functional output. The dispensability of the SYG-1 cytoplasmic domain and the rescue produced by synthetic adhesion argue that a specialized downstream signaling program is not required for substantial restoration of branch orientation. While members of the Neph and Robo families have been extensively characterized as signaling receptors in axon guidance and epithelial organization [22, 25, 26], emerging studies suggest that these proteins can also contribute to structural integrity in mechanically stressed environments, such as the slit diaphragm in podocytes [44]. Our findings thus extend this concept by showing that membrane-tethered extracellular interactions can substantially stabilize neurite position *in vivo* under physiological tissue deformation. This distinction expands current paradigms of neurite morphogenesis by demonstrating that an extracellular adhesive linkage can be sufficient to substantially restore branch positioning in this context.

Ligands acting as molecular “matchmakers” to promote receptor complex formation have been described in multiple systems. In *Drosophila*, cleaved Slit fragments enhance the association between Robo1 and Dscam1 to promote axon growth [38]. In *C. elegans*, the co-receptor EVA-1 facilitates SLT-1/SAX-3 interactions to tune signaling sensitivity [45]. Similarly, co-receptors and extracellular scaffolds, such as heparan sulfate proteoglycans, can facilitate ligand-receptor clustering and stabilize signaling complexes in guidance pathways [46, 47]. In our study, we propose that SLT-1 functions analogously as a structural facilitator, promoting the formation of a transcellular SYG-1/SAX-3 adhesive interface rather than acting as an instructive guidance cue. Determining how SLT-1 promotes this transcellular interaction, and whether it directly contacts both receptors, will require further structural and biochemical analysis.

The incomplete penetrance of the *sax-3* and *slt-1* phenotypes relative to *syg-1* further suggests that this anchoring mechanism may not correspond to a strictly obligate SYG-1–SLT-1–SAX-3 complex. Loss of SYG-1 produces a substantially stronger branch-orientation defect than loss of SAX-3 or SLT-1, despite the lack of obvious enhancement in the corresponding double mutants. One possibility is that SYG-1 represents the principal neuronal anchoring component, whereas SAX-3 and SLT-1 facilitate only a subset of SYG-1-dependent adhesive interactions. Additional epithelial ligands or extracellular factors may therefore cooperate with SYG-1 in parallel with the SAX-3/SLT-1 module. Alternatively, residual SAX-3-independent adhesion or differences in the effective loss of function of the available alleles could contribute to the penetrance difference. Thus, our data are most consistent with SLT-1 and SAX-3 promoting or stabilizing the SYG-1-dependent adhesive interface, rather than being absolutely required for every SYG-1-mediated anchoring event.

Together, our findings show that precise neural architecture depends not only on instructive signals that establish neurite trajectories, but also on mechanisms that preserve those trajectories as tissues subsequently grow and deform. The SYG-1/SAX-3 system illustrates how classical guidance and adhesion receptors can be repurposed to provide this structural resilience. Such transcellular adhesive tethers may represent a broader strategy for maintaining circuit organization in mechanically dynamic tissues.

### Methods

#### C. elegans strains

*C. elegans* strains were cultured and maintained as previously described [48]. In brief, animals were cultivated on nematode growth medium (NGM) plates seeded with *E. coli* OP50 bacteria at 20°C. All strains used in the study are listed in supplementary Table 1.

## Molecular Cloning

Promoter fragments were PCR-amplified from *C. elegans* genomic DNA and cloned into the pPD95.75 vector for tissue-specific expression: *ocr-3* promoter (0.5 kb) for PVP interneuron labeling, *unc-119* promoter (1.6 kb) for pan-neuronal expression, *nlp-3* promoter (3.8 kb) for HSN motor neuron expression, *daf-6* promoter (3.5 kb) for vulval E and F cell expression, *hsp-16.2* promoter (0.4 kb) for heat-shock inducible expression, *ceh-23L* promoter (0.6 kb) for CAN neuron expression, and *slt-1* promoter (4.5 kb) for functional rescue experiments. Full-length cDNAs of *syg-1* and *sax-3* were amplified from a cDNA library prepared using the SuperScript III First-Strand Synthesis System (ThermoFisher, Cat#18080051) and cloned downstream of the indicated promoters in pPD95.75-based expression vectors. All primers used for constructs are listed in Supplementary Table 2.

The GFP (enhanced GFP) and anti-GFP nanobody (LaG16, Kd = 0.7 nM) sequences were obtained from published work [39, 40]. For the PVP neuron construct, the anti-GFP nanobody coding sequence was fused to a CD4 transmembrane domain and cloned downstream of the *ocr-3* promoter (0.5 kb) in a pPD95.75-based vector. For the vulva cell construct, GFP was similarly fused to a CD4 transmembrane domain and cloned downstream of the *daf-6* promoter (3.5 kb) to drive expression in vulval E and F cells. No intracellular signaling domains were included in either construct.

To generate constructs for the S2 cell aggregation assay, SYG-1 and SAX-3 cDNAs lacking their intracellular domains (ΔC) were cloned into the pAc5.1 vector (Ac5 promoter, 2.5 kb) and fused in-frame to NeonGreen or mKate2, respectively. Full-length SLT-1 genomic DNA was cloned into pAc5.1 to generate an SLT-1 expression construct for producing SLT-1-conditioned medium.

## Germline Transformation

Germline transformation was performed by microinjecting plasmids in gonads as described [49]. In brief, the gonads of D1 adult worms were visualized by an inverted microscope (Zeiss, Axio Observer) at 40X objective, and the DNA mixture was injected through a microinjector (Eppendorf FemtoJet 4X). Isolated progeny carrying transgenes for more than two generations were considered independent transgenic lines for experiments.

## FUdR Treatment

30–50 D1 adult animals were quantified by picking L4 larvae one day before scoring.

A 5 mM FUdR (Sigma-Aldrich, Cat. F0503) working solution was prepared in M9, and 100 µL was spread onto OP50-seeded NGM plates and culturing overnight at 20°C. Adult scored adult animals the following day. At the time of scoring, FUdR-treated animals were further classified according to whether eggs were present in the reproductive tract.

## Image Acquisition

Confocal fluorescent images were acquired by LSM 780 Confocal microscopy (LSM 780, Zeiss). Epifluorescent images were acquired by a Zeiss Imager M2 microscope with an Axiocam 705 Mono camera. Z-stack epifluorescent images were acquired by a Zeiss Imager M2 microscope equipped with an Apotome 3 system. Images were analyzed using ZEN Blue 3.6 software (Zeiss).

## Developmental imaging and quantification of LifeAct signals

PVP branch development and F-actin organization were examined using animals expressing PVP-specific fluorescent reporters. Animals at the indicated developmental stages were mounted on 5% agarose pads and paralyzed with 1% sodium azide or 10 mM levamisole (time-lapse imaging). PVP morphology and LifeAct signals were examined at successive developmental stages from late larval development to adulthood to determine the initial direction and subsequent positioning of individual PVP branches.

For quantification of F-actin enrichment, LifeAct fluorescence intensity was measured using the line-scan function in ImageJ as previously described. The vulval slit or P6.p-derived vulval cells were used as the midpoint, and fluorescence intensity was measured along the PVP process over a 30-μm region extending 15 μm anterior and posterior to the midpoint. The central 5-μm region was defined as the vulval region, and the remaining 25 μm was defined as the non-vulval region. F-actin enrichment was expressed as the ratio of the average LifeAct fluorescence intensity within the vulval region to that of the non-vulval region.

## Quantification of reversed PVP branching

30–50 D1 adult animals were quantified by picking L4 larvae a day before scoring. Animals were then mounted on a 5% agarose pad and paralyzed with 1% sodium azide. Images were taken by ZEN Blue 3.6 software using a 63X objective in a Zeiss Imager M2 microscope with an Axiocam 705 Mono camera. A mature branch was defined as a protrusion > 5 μm in length. A branch was classified as reversed when it projected ventrally from the longitudinal PVP process. The percentage of PVP branch projection in a population was subsequently scored.

## Tissue-specific expression and tissue swapping

Tissue-specific expression constructs were generated using the promoters described above. For reciprocal tissue-swapping experiments, cytoplasmic-domain-deleted SYG-1 (SYG-1ΔC) was expressed in vulval epithelial cells under the *daf-6* promoter, whereas cytoplasmic-domain-deleted SAX-3 (SAX-3ΔC) was expressed in PVP neurons under the *ocr-3* promoter. The corresponding constructs were introduced into the indicated mutant backgrounds by germline transformation, and independent transgenic lines were analyzed for PVP branch orientation.

## S2 Cell Aggregation Assay

*Drosophila Schneider 2 (S2)* cells were transfected using Effectene (QIAGEN). Separate cell populations were transfected with either SAX-3ΔC::mKate or SYG-1ΔC::NeonGreen. To generate SLT-1-conditioned medium, S2 cells were transfected with an SLT-1 expression construct, while untransfected S2 cells were used to prepare control conditioned medium. After three days of culture, SYG-1ΔC- and SAX-3ΔC-expressing cells were enriched by fluorescence-activated cell sorting using a FACSAria system (BD Biosciences, San Jose, CA), yielding populations with > 99% fluorescently positive cells.

Sorted cells were washed twice with phosphate-buffered saline (PBS), resuspended in S2 medium, and adjusted to a density of $2 \times 10^7$ cells/ml. Equal populations of SYG-1ΔC- and SAX-3ΔC-expressing cells were mixed and incubated in 48-well plates in the presence of either control or SLT-1-conditioned medium. Cell suspensions were rotated at 120 rpm for 1 h at room temperature. An aliquot of 10 μl was then transferred to a glass slide and imaged using a 10x objective on a Zeiss Axio Imager M2 microscope. Higher-resolution images were acquired using a Zeiss LSM700 confocal microscope. Cell aggregates were defined as clusters containing colocalized SYG-1ΔC::NeonGreen- and SAX-3ΔC::mKate-expressing cells. Aggregation was quantified as the percentage of cells incorporated into aggregates relative to the total number of cells analyzed.

## Morphology-linked egg-laying assay

Individual D1 adult hermaphrodites were placed separately into wells containing M9 buffer with 18.5 mM serotonin hydrochloride (Sigma-Aldrich, Cat. H9523), and the number of eggs laid by each animal was counted after 1 h. For mock controls, animals were placed in M9 buffer without serotonin. Immediately following the behavioral assay, each animal was recovered individually and subsequently examined for PVP branch orientation. Animals were classified according to whether the PVP branch exhibited correct dorsal targeting or reversed ventral targeting. Egg-laying numbers obtained before morphological examination were then grouped according to the subsequently determined PVP branch position for statistical comparison.

## Statistical analysis

Statistical analyses were performed using GraphPad Prism (version 10.4.1). Data were analyzed using one-way analysis of variance (ANOVA) followed by Tukey's multiple-comparisons test, two-tailed unpaired Student's *t*-test, or Fisher's exact test, as indicated in the corresponding figure legends. Statistical significance was defined as $P < 0.05$. Exact $P$ values are reported in the figures or figure legends where applicable. For categorical analyses of PVP branch orientation, the number of animals exhibiting correctly oriented or reversed branches was compared using Fisher's exact test. For continuous measurements, including fluorescence-based quantification, cell-aggregation measurements, and egg-laying assays, comparisons between two groups were performed using two-tailed unpaired Student's *t*-tests, whereas comparisons involving more than two groups were performed using one-way ANOVA followed by Tukey's multiple-comparisons test. Sample sizes ($n$) represent individual animals or independent experimental samples, as specified in the corresponding figure legends.

### Declarations

## Materials availability

Plasmids and strains generated in this study are available upon request.

## Data Availability

All data were included in the article and/or supplementary information. All raw data were deposited to Figshare (private link: https://figshare.com/s/995772e2a93fd940a0c7).

The Figshare dataset will be made publicly available upon publication.

## Competing interests

The authors declare no competing interests.

**Declarations of Generative AI and AI-assisted Technologies in the Writing Process**

During the preparation of this work, the authors used ChatGPT to improve the language, grammar, and readability of the manuscript. After using this tool/service, the authors reviewed and edited the content as needed and take full responsibility for the content and integrity of the final publication.

## Author Contributions

Y.-H.L. and C.-H.C. conceived the study and designed the experiments. Y.-H.L. performed the experiments and analyzed the data. Y.-H.L. and C.-H.C. interpreted the results and wrote the manuscript. C.-H.C. supervised the project and acquired funding.

## Acknowledgments

We thank Dr. Chun-Liang Pan, Dr. Han Wang, and Dr. Byunghyuk Kim, and members of the Chen lab for their insightful discussion. We also thank Dr. Chun-Liang Pan for providing reagents. Some strains were provided by the *C. elegans* Genetics Center, funded by the NIH Office of Research Infrastructure Programs (P40 OD010440). We are grateful for the technical assistance of Technology Commons, the College of Life Science, and the National Taiwan University with confocal microscopy (CLSM). We would also like to acknowledge the service provided by the Flow Cytometric Analyzing and Sorting Core of the First Core Laboratory, National Taiwan University College of Medicine. We thank the *C. elegans* Core Facility of the National Core Facility for Biopharmaceuticals, Ministry of Science of Technology in Taiwan. This study was funded by the National Science and Technology Council (R.O.C), Taiwan, to C.-H.C. (NSTC 112-2636-B-002-007, NSTC 113-2636-B-002-003, NSTC 114-2311-B-002-024).

## Figures

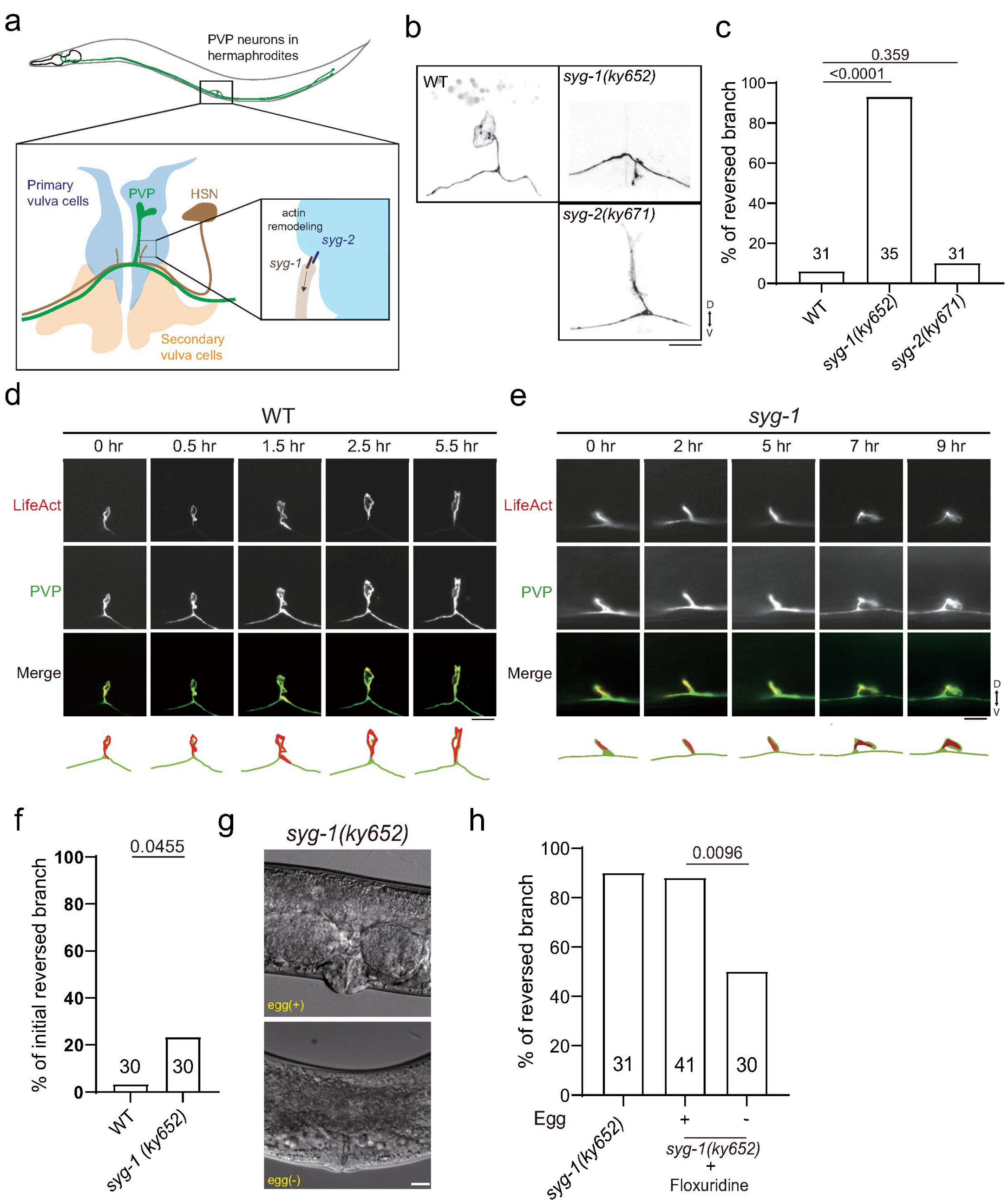

### Figure 1

**SYG-1 maintains PVP branch orientation against reproductive tissue deformation.**

a, Schematic of the sexually dimorphic PVP collateral branch adjacent to the vulva and the canonical SYG-1/SYG-2-dependent interaction between HSN and primary vulval epithelial cells. b, Representative confocal images of PVP branches in wild-type (WT), *syg-1(ky652)*and *syg-2(ky671)* hermaphrodites.

Scale bar= 5 µm. c, Percentage of animals exhibiting reversed, ventrally oriented PVP branches. Loss of *syg-1*, but not *syg-2*, strongly disrupts branch orientation. d,e, Representative developmental time series showing LifeAct-labelled F-actin (red), PVP morphology (green) and merged images in WT (d) and *syg-1(ky652)* (e) animals. Schematics below illustrate branch orientation over time. WT branches remain dorsally oriented, whereas initially dorsal branches in *syg-1*mutants can subsequently become displaced ventrally. LifeAct enrichment persists at the branch site during this displacement. Scale bar= 5 µm. f, Percentage of PVP branches already exhibiting reversed orientation at the initial imaging time point, demonstrating a contribution of SYG-1 to initial directional bias. g, Representative *syg-1(ky652)* animals following floxuridine (FUdR) treatment, classified according to the presence [egg(+)] or absence [egg(-)] of eggs in the reproductive tract. Scale bar= 10 µm. h, Quantification of reversed branches in untreated and FUdR-treated *syg-1(ky652)*animals. The absence of eggs in FUdR-treated animals is associated with reduced branch reversal. Numbers indicate animals analyzed. Statistical comparisons were performed using two-sided Fisher's exact test; exact *P* values are shown.

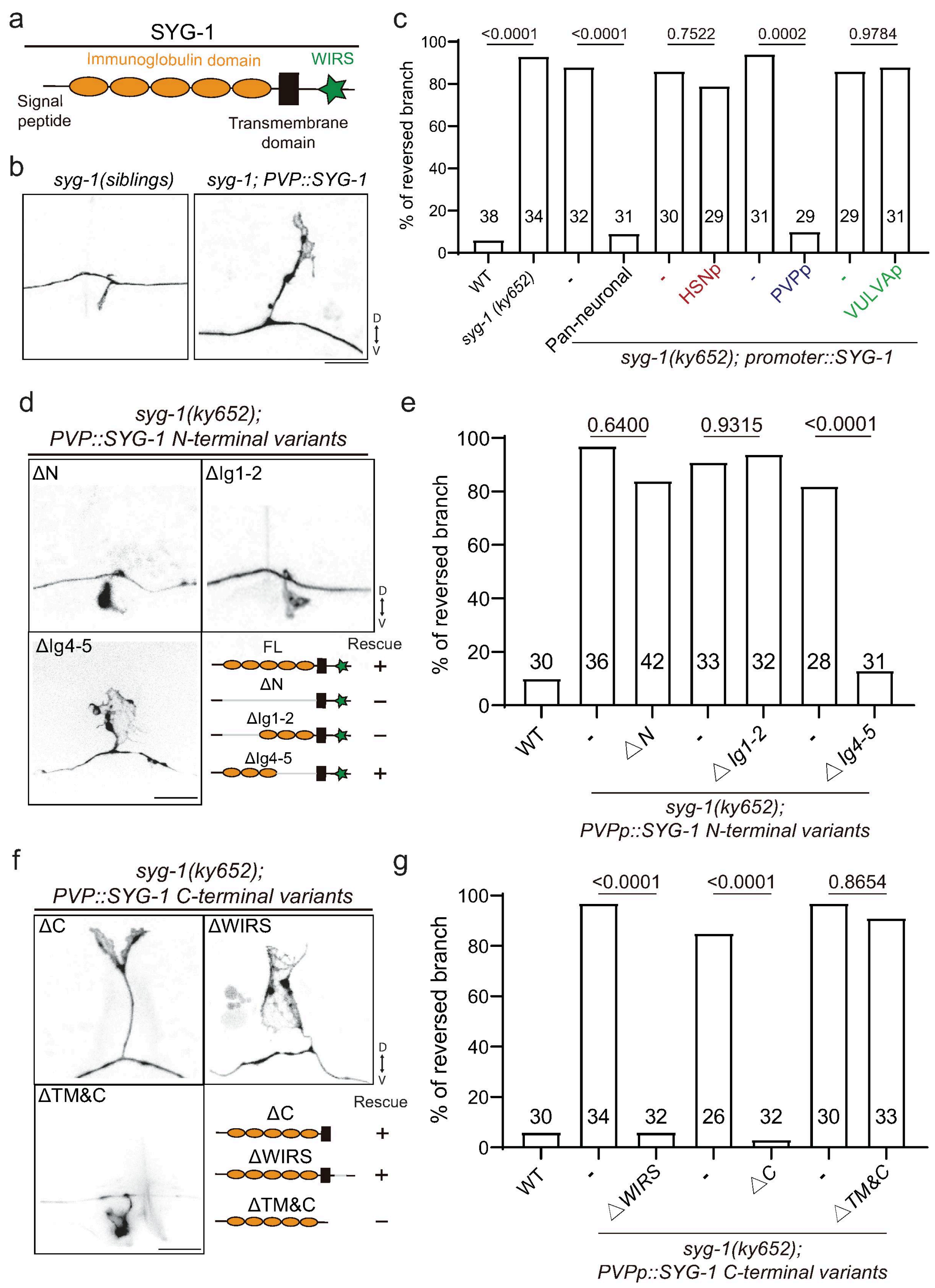

**Figure 2**

**SYG-1 functions cell-autonomously through its membrane-tethered extracellular domain.**

a, Domain organization of SYG-1, showing the N-terminal signal peptide, five extracellular immunoglobulin (Ig) domains, transmembrane domain and cytoplasmic WIRS motif. b, Representative confocal images of PVP morphology in *syg-1* control siblings and *syg-1(ky652)*animals expressing fulllength SYG-1 specifically in PVP neurons. Scale bar= 5 μm. c, Tissue-specific rescue of the reversed-branch phenotype. Pan-neuronal or PVP-specific expression of SYG-1 restores correct branch orientation, whereas expression in HSN neurons or vulval epithelial cells does not. d, Representative confocal images of PVP branches and schematics of SYG-1 extracellular-domain deletion variants expressed specifically in PVP neurons. Scale bar= 5 μm. e, Quantification of branch orientation following expression of SYG-1 variants lacking the entire N-terminal extracellular region (ΔN), Ig domains 1-2 (ΔIg1-2) or Ig domains 4-5 (ΔIg4-5). ΔN and ΔIg1-2 abolish rescue, whereas ΔIg4-5 retains rescue activity. f, Representative confocal images of PVP and schematics of SYG-1 variants lacking the cytoplasmic domain (ΔC), WIRS motif (ΔWIRS) or both the transmembrane and cytoplasmic domains (ΔTM&C). Scale bar= 5 μm. g, Quantification of branch orientation following expression of the indicated variants. ΔC and ΔWIRS retain rescue activity, whereas ΔTM&C does not. Numbers indicate animals analyzed. Statistical comparisons were performed using two-sided Fisher's exact test; exact *P* values are shown.

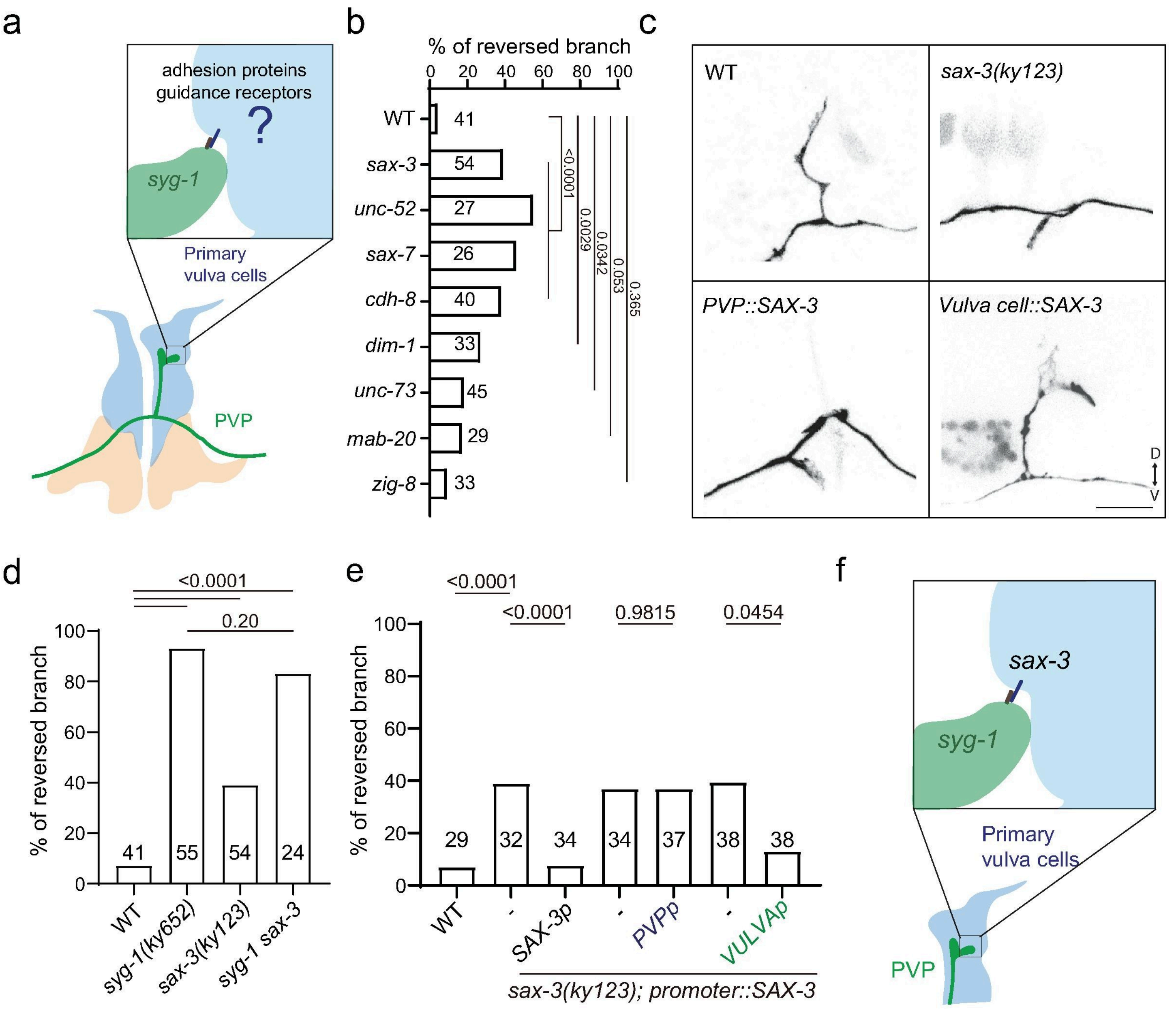

**Figure 3**

**SAX-3/Robo functions in vulval epithelial cells in the same genetic pathway as SYG-1.**

a, Schematic of the candidate screen for vulval-expressed adhesion molecules and guidance receptors that could function across the PVP-vulval interface with neuronal SYG-1. b, Percentage of animals exhibiting reversed PVP branches in WT and the indicated candidate mutants. Loss of *sax-3* produces a strong reversed-branch phenotype. c, Representative confocal images of PVP morphology in WT and *sax-3(ky123)* animals and in *sax-3* mutants expressing SAX-3 specifically in PVP neurons or vulval epithelial cells. Scale bar= 5 μm. d, Quantification of reversed PVP branches in WT, *syg-1(ky652)*, *sax-3(ky123)* and *syg-1 sax-3* double mutants. The double mutant shows no enhancement relative to the single mutants, consistent with *syg-1* and *sax-3* acting in the same genetic pathway. e, Tissue-specific rescue of *sax-3(ky123)* animals. Expression of SAX-3 from its endogenous promoter or specifically in vulval epithelial cells restores branch orientation, whereas PVP-specific expression does not. f, Model in which neuronal SYG-1 and vulval epithelial SAX-3 function across the neuron-epithelium interface to stabilize PVP branch orientation. Numbers indicate animals analyzed. Statistical comparisons were performed using two-sided Fisher's exact test; exact *P* values are shown.

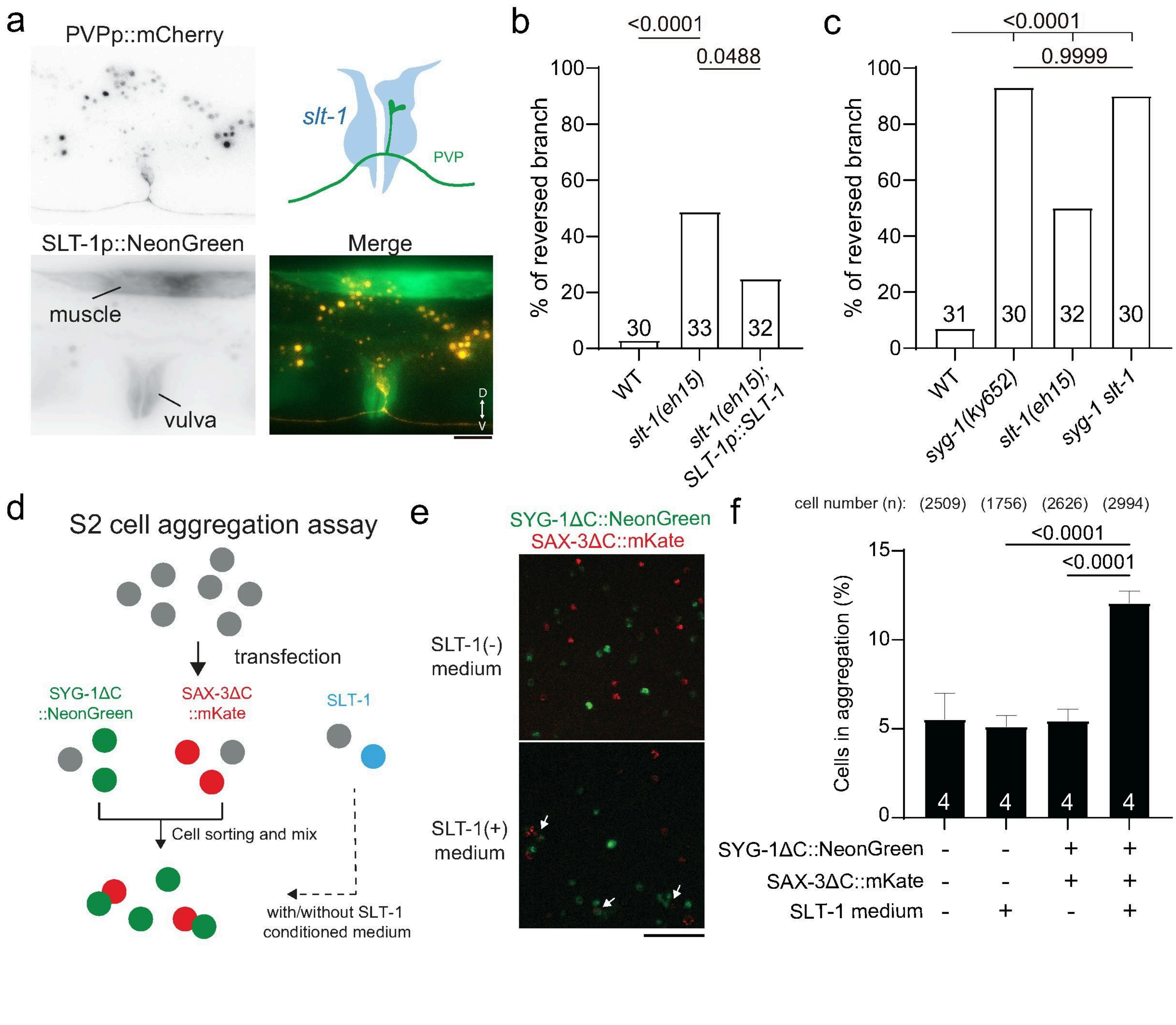

**Figure 4**

**SLT-1 promotes SYG-1/SAX-3-dependent extracellular adhesion.**

a, Expression of *slt-1* in the vulval region. Representative epifluorescence images show PVP morphology (PVPp::mCherry), *slt-1* promoter activity (SLT-1p::NeonGreen), and the merged image; the schematic illustrates *slt-1* expression relative to PVP and vulval cells. Scale bar= 10 μm. b, Percentage of reversed PVP branches in WT, *slt-1(eh15)*and *slt-1(eh15)* animals carrying an endogenous-promoter SLT-1 rescue construct. c, Quantification of reversed PVP branches in WT, *syg-1(ky652)*, *slt-1(eh15)* and *syg-1 slt-1* double mutants. Lack of enhancement in the double mutant is consistent with SLT-1 and SYG-1 functioning in the same pathway. d, Schematic of the S2-cell aggregation assay. Cells separately expressing cytoplasmic-domain-deleted SYG-1 (SYG-1ΔC::NeonGreen) or SAX-3 (SAX-3ΔC::mKate) were sorted, mixed and incubated with control or SLT-1-conditioned medium. e, Representative epifluorescence images of mixed SYG-1ΔC- and SAX-3ΔC-expressing S2 cells incubated with control [SLT-1(−)] or SLT-1-conditioned [SLT-1(+)] medium. Scale bar= 50 mm. Arrows indicate mixed-cell aggregates. f, Percentage of cells incorporated into aggregates under the indicated conditions. Numbers within bars indicate independent transfection and aggregation experiments. Data are mean ± s.e.m. Categorical branch-orientation data were analyzed using two-sided Fisher's exact test; cell-aggregation data were analyzed by one-way ANOVA followed by Tukey's multiple-comparisons test. Exact *P* values are shown.

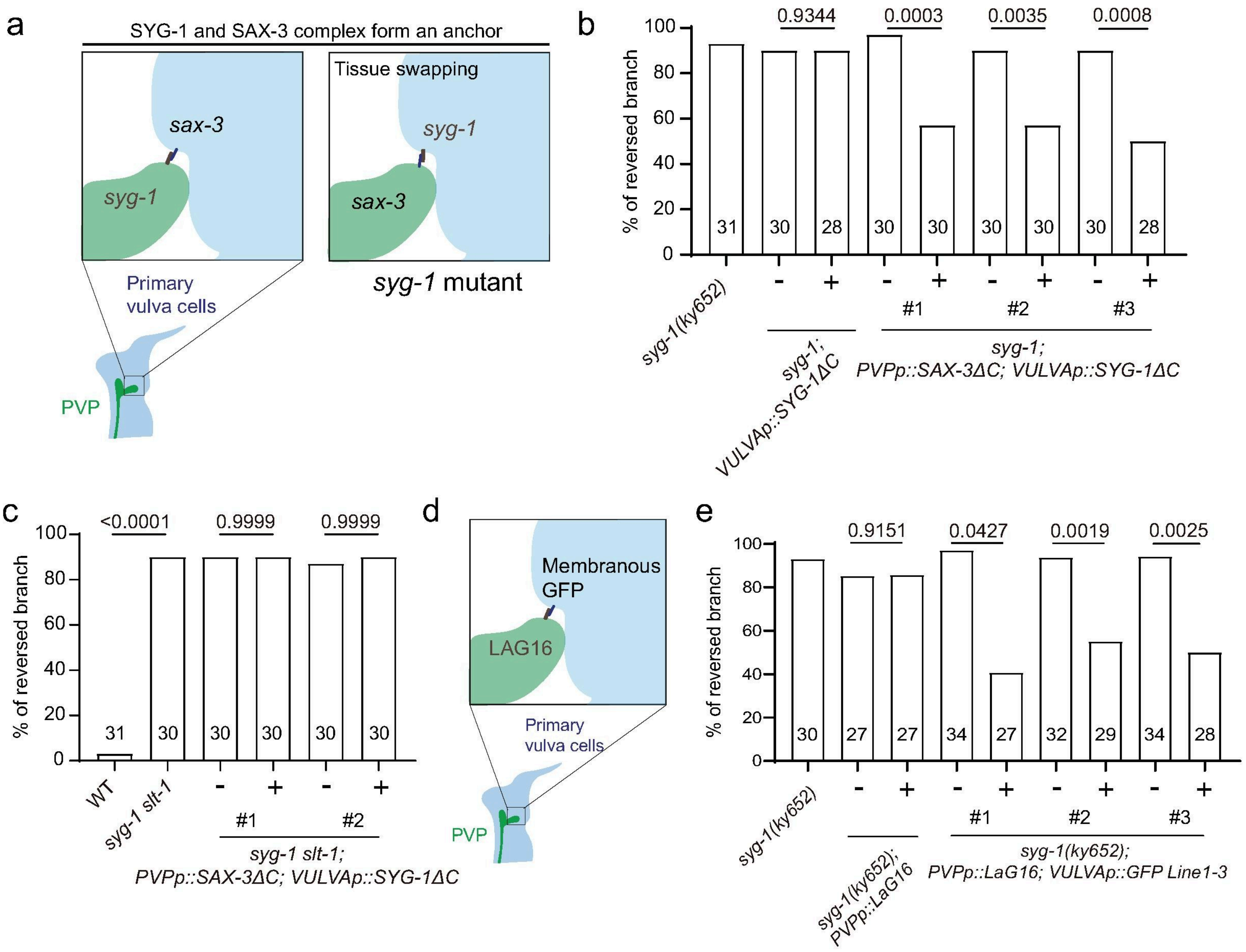

**Figure 5**

**Synthetic extracellular adhesion partially restores PVP branch orientation.**

a, Schematic of the native and tissue-swapped SYG-1/SAX-3 configurations. In the native configuration, SYG-1 is present in PVP neurons and SAX-3 in opposing vulval epithelial cells. In the tissue-swapping experiment, cytoplasmic-domain-deleted SAX-3 (SAX-3ΔC) was expressed in PVP neurons and SYG-1ΔC in vulval epithelial cells. b, Quantification of reversed PVP branches following reciprocal expression of PVPp::SAX-3ΔC and VULVAp::SYG-1ΔC in *syg-1(ky652)* animals. Three independent transgenic lines (#1–#3) are shown. c, Requirement for SLT-1 in rescue by the tissue-swapped SYG-1ΔC/SAX-3ΔC pair. Reciprocal expression fails to restore branch orientation in *syg-1 slt-1* animals. Two independent transgenic lines are shown. d, Schematic of the synthetic adhesion system in which membrane-tethered anti-GFP nanobody LaG16 is expressed in PVP neurons and membrane-tethered GFP in adjacent vulval epithelial cells. e, Quantification of reversed branches in *syg-1(ky652)* animals carrying the synthetic adhesion system. PVP-specific LaG16 alone does not rescue branch orientation, whereas co-expression of PVPp::LaG16 and vulval membrane-tethered GFP significantly reduces branch reversal in three independent transgenic lines. Numbers indicate animals analyzed. Statistical comparisons were performed using two-sided Fisher's exact test; exact *P* values are shown.

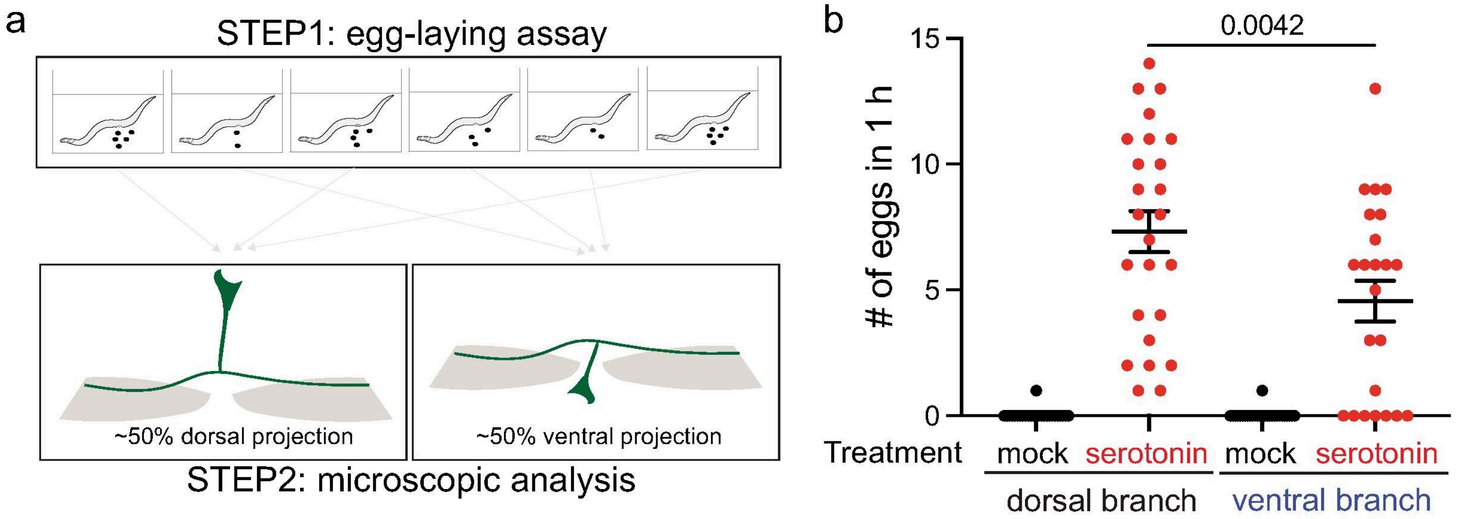

**Figure 6**

**Correct PVP branch orientation is associated with efficient egg laying.**

a, Experimental design linking egg-laying behavior to PVP branch morphology in individual animals. Egg laying was first quantified for 1 h under mock or serotonin-treated conditions, after which each animal was recovered and examined microscopically and classified as having a correctly targeted dorsal or reversed ventral PVP branch. b, Number of eggs laid during 1 h by animals subsequently classified according to PVP branch orientation. Under serotonin stimulation, animals with dorsally targeted PVP branches lay significantly more eggs than animals with ventrally reversed branches. Each dot represents one animal; horizontal bars indicate mean ± s.e.m. One-way ANOVA followed by Tukey's multiple-comparisons test. Exact *P* value is shown.

## Supplementary Files

This is a list of supplementary files associated with this preprint. Click to download.

- SupplementaryFigurecombined.pdf
- SupplementaryTable120260917.xlsx
- SupplementaryTable220260917.xlsx
